# Supplementary material for: Contribution of “complete response to treatment” to survival in patients with unresectable metastatic colorectal cancer: A retrospective analysis
Source: PLoS One. 2021 Nov 8;16(11):e0259622. doi: 10.1371/journal.pone.0259622 (PMC8575296; doi:10.1371/journal.pone.0259622)
Supplement: S1 File — (DOCX) [file pone.0259622.s001.docx]

**TÜM OLGULAR**

**Kategorik Veriler**

|  |  | **Frequency** | **Percent** |
| --- | --- | --- | --- |
| Cinsiyet | Erkek | 134 | 60,9 |
|  | Kadın | 86 | 39,1 |
| Primer bölge | Rektum | 82 | 37,6 |
|  | Distal kolon | 81 | 37,2 |
|  | Proksimal kolon | 44 | 20,2 |
|  | Transvers kolo | 11 | 5 |
| Primer cerrahi | Yok | 82 | 38 |
|  | Var | 132 | 61,1 |
| Histoloji | Bilinmiyor | 11 | 5 |
|  | Adeno ca | 184 | 83,6 |
|  | Müsinöz adeno ca | 19 | 8,6 |
|  | Taşlı yüzük adeno ca | 3 | 1,4 |
|  | Nöroendokrin dif, Adeno ca | 3 | 1,4 |
| Diferansiasyon | İyi | 11 | 5 |
|  | Orta | 98 | 44,5 |
|  | Kötü | 17 | 7,7 |
|  | Bilinmiyor | 94 | 42,7 |
| T | T1 | 1 | 0,7 |
|  | T2 | 1 | 0,7 |
|  | T3 | 69 | 47,3 |
|  | T4 | 48 | 32,9 |
|  | Tx | 27 | 18,5 |
| LVI | Var | 51 | 71,8 |
|  | Yok | 20 | 28,2 |
| PNI | Var | 39 | 63,9 |
|  | Yok | 22 | 36,1 |
| KRAS durumu | Bilinmiyor | 79 | 35,9 |
|  | Mutant | 65 | 29,5 |
|  | Wild tip | 76 | 34,5 |
| Metastaz zamanı | Tanı anı | 163 | 75,5 |
|  | Adjuvan tedavi sonrası | 53 | 24,5 |
| KC metastazı | Yok | 78 | 35,5 |
|  | Var | 142 | 64,5 |
| Kemik metastazı | Yok | 198 | 90 |
|  | Var | 22 | 10 |
| Neoadjuvan tedavi | Yok | 6 | 50 |
|  | Var | 6 | 50 |
| Adjuvan tedavi | Yok | 171 | 78,1 |
|  | Var | 48 | 21,9 |
| Tadavi ile tam yanıt | Tam yanıt var | 31 | 14,6 |
|  | Tam yanıt yok | 181 | 85,4 |
| Progresyon | Yok | 4 | 2,3 |
|  | Var | 172 | 97,7 |
| Ölüm | Yaşıyor | 18 | 8,2 |
|  | Ex | 202 | 91,8 |

**SAYISAL VERİLER**

|  | **N** | **Minimum** | **Maximum** | **Mean** | **Std, Deviation** |
| --- | --- | --- | --- | --- | --- |
| Tanı yaşı | 219 | 24,8 | 83,9 | 60,13 | 12,523 |
| Metastazsız interval (ay) | 215 | 0 | 100,3 | 5,933 | 12,8163 |

|  | **N** | **Minimum** | **Maximum** | **Median** | **Mean** | **%95 CI)** |
| --- | --- | --- | --- | --- | --- | --- |
| Metastatik bölge sayısı | 220 | 1 | 6 | 1 | 1,69 | 1,57-1,81 |

**TEDAVİYE VERİLEN YANITA GÖRE ALT GRUPLAR (Karşılaştırma)**

**Kategorik Veriler (Chi-Square Tests)**

|  | | **Tam yanıt var** | | **Tam yanıt yok** | | **p** |
| --- | --- | --- | --- | --- | --- | --- |
|  |  | **Frequency** | **Percent** | **Frequency** | **Percent** |  |
| Cinsiyet | Erkek | 18 | 0,14 | 111 | 0,86 | 0,731 |
|  | Kadın | 13 | 0,157 | 70 | 0,843 |  |
| Primer bölge | Rektum | 7 | 0,089 | 72 | 0,911 | 0,160 |
|  | Distal kolon | 15 | 0,19 | 64 | 0,81 |  |
|  | Proksimal kolon | 6 | 0,143 | 36 | 0,857 |  |
|  | Transvers kolo | 3 | 0,3 | 7 | 0,7 |  |
| **Primer cerrahi** | **Yok** | **3** | **0,038** | **77** | **0,963** | **0,000** |
|  | **Var** | **28** | **0,219** | **100** | **0,781** |  |
| Histoloji | Bilinmiyor | 1 | 0,091 | 10 | 0,909 | 0,364 |
|  | Adeno ca | 25 | 0,14 | 153 | 0,86 |  |
|  | Müsinöz adeno ca | 5 | 0,294 | 12 | 0,706 |  |
|  | Taşlı yüzük adeno ca | 0 | 0 | 3 | 1 |  |
|  | Nöroendokrin dif, Adeno ca | 0 | 0 | 3 | 1 |  |
| Diferansizasyon | İyi | 2 | 0,182 | 9 | 0,818 | 0,460 |
|  | Orta | 21 | 0,216 | 76 | 0,784 |  |
|  | Kötü | 1 | 0,063 | 15 | 0,938 |  |
|  | Bilinmiyor | 7 | 0,08 | 81 | 0,92 |  |
| T | T1 | 1 | 1 | 0 | 0 | - |
|  | T2 | 1 | 1 | 0 | 0 |  |
|  | T3 | 16 | 0,25 | 48 | 0,75 |  |
|  | T4 | 9 | 0,191 | 38 | 0,809 |  |
|  | Tx | 0 | 0 | 26 | 1 |  |
| LVI | Var | 13 | 0,26 | 37 | 0,74 | 0,303 |
|  | Yok | 7 | 0,389 | 11 | 0,611 |  |
| PNI | Var | 9 | 0,243 | 28 | 0,757 | 0,461 |
|  | Yok | 7 | 0,333 | 14 | 0,667 |  |
| KRAS durumu | Mutant | 10 | 0,159 | 53 | 0,841 | 0,759 |
|  | Wild tip | 12 | 0,16 | 63 | 0,84 |  |
| Metastaz zamanı | Tanı anı | 24 | 0,153 | 133 | 0,847 | 0,786 |
|  | Adjuvan tedavi sonrası | 7 | 0,137 | 44 | 0,863 |  |
| **KC metastazı** | **Yok** | **16** | **0,213** | **59** | **0,787** | **0,041** |
|  | **Var** | **15** | **0,109** | **122** | **0,891** |  |
| **Kemik metastazı** | **Yok** | **31** | **0,162** | **160** | **0,838** | **0,046** |
|  | **Var** | **0** | **0** | **21** | **1** |  |
| Adjuvan tedavi | Yok | 23 | 0,14 | 141 | 0,86 | 0,609 |
|  | Var | 8 | 0,17 | 39 | 0,83 |  |
| Birinci sıra tedavi | Fluoropyrimidine | 1 | 5,6 | 17 | 94,4 | 0,276 |
|  | Irinotecan | 12 | 19,4 | 50 | 80,6 |  |
|  | Oxaliplatin | 17 | 13,1 | 113 | 86,9 |  |
| **Exitus** | **Hayır** | **9** | **0,529** | **8** | **0,471** | **0,000** |
|  | **Ex** | **22** | **0,113** | **173** | **0,887** |  |

**Sayısal Veriler**

(Student T Test)

|  |  | **N** | **Mean** | **Std, Deviation** | **P** |
| --- | --- | --- | --- | --- | --- |
| Tanı yaşı | Tam yanıt yok | 180 | 60,5 | 12,2 | 0,192 |
|  | Tam yanıt var | 31 | 57,3 | 12,6 |  |
| Metastazsız interval (ay) | Tam yanıt yok | 176 | 5,8 | 13,2 | 0,767 |
|  | Tam yanıt var | 31 | 6,5 | 11,7 |  |

(Mann-Whitney U Test)

|  |  | N | Median | %95 CI | P |
| --- | --- | --- | --- | --- | --- |
| Metastatik bölge sayısı | Tam yanıt yok | 181 | 1 | 1,60-1,87 | - |
|  | Tam yanıt var | 31 | 1 | 1,09-1,62 |  |

**ÇOK DEĞİŞKENLİ ANALİZ (Logistic regression) - Değişkenlerin “tedaviye tam yanıt” alınmasına etkileri**

|  | Exp(B) | 95% C.I.for EXP(B) | | Sig. |
| --- | --- | --- | --- | --- |
|  |  | Lower | Upper |  |
| Tanı yaşı | .990 | .958 | 1.024 | .577 |
| **Primer cerrahi** | **.168** | **.047** | **.594** | **.006** |
| KC metastazı | 1.655 | .723 | 3.790 | .234 |
| Kemik metastazı | 302.766.813.049 | .000 | . | .998 |
| Fluoropyrimidine |  |  |  | .938 |
| Irinotecan | .687 | .076 | 6.217 | .738 |
| Oxaliplatin | 1.042 | .436 | 2.486 | .927 |

**SAĞKALIM ANALİZİ (Kaplan-Meier)**

| **OS** | **Medyan (ay)** | **%95 güven aralığı** | | **P** |
| --- | --- | --- | --- | --- |
|  |  | **Alt sınır** | **Üst sınır** |  |
| Tam yanıt yok | 16.990 | 14.702 | 19.278 |  |
| Tam yanıt var | 39.230 | 22.141 | 56.319 | <0,001 |
| Toplam | 19.580 | 16.646 | 22.514 |  |


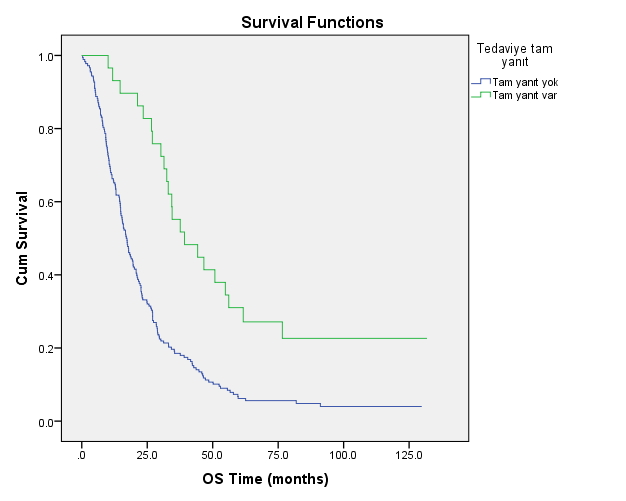


| **PFS** | **Medyan (ay)** | **%95 güven aralığı** | | **P** |
| --- | --- | --- | --- | --- |
|  |  | **Alt sınır** | **Üst sınır** |  |
| Tam yanıt yok | 7.490 | 6.088 | 8.892 |  |
| Tam yanıt var | 15.240 | 13.126 | 17.354 | <0,001 |
| Toplam | 8.800 | 7.422 | 10.178 |  |


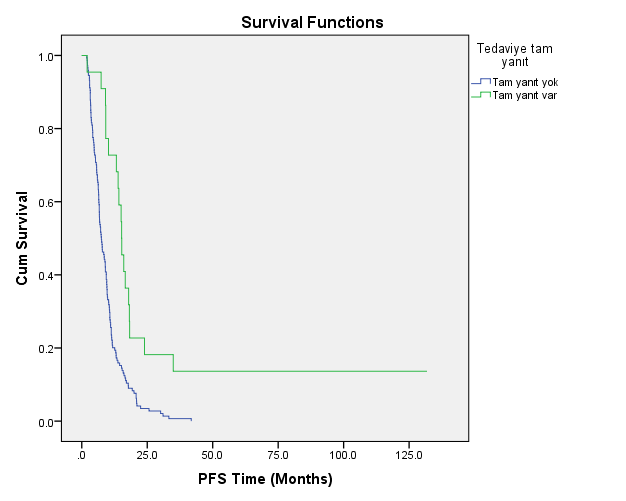


**TEK DEĞİŞKENLİ ANALİZ (Cox regresyon analizi) - Değişkenlerin sağkalıma etkisi**

|  | **Exp(B)** | **95.0% CI for Exp(B)** | | **Sig.** |
| --- | --- | --- | --- | --- |
|  |  | **Lower** | **Upper** |  |
| Tanı yaşı | 1.007 | .995 | 1.019 | .265 |
| Cinsiyet (Kadına göre erkek) | .824 | .618 | 1.100 | .190 |
| Primer bölge (Rektum) |  |  |  | .726 |
| Primer bölge (Distal kolon) | .690 | .367 | 1.300 | .252 |
| Primer bölge (Proksimal kolon) | .722 | .382 | 1.365 | .316 |
| Primer bölge (Transters kolon) | .724 | .370 | 1.416 | .345 |
| **Primer cerrahi (Uygulanmayana göre uygulanan)** | **1.719** | **1.282** | **2.305** | **.000** |
| Histoloji (Adeno) |  |  |  | .178 |
| Histoloji (Müsinöz) | .470 | .149 | 1.488 | .199 |
| Histoloji (Taşlı yüzük) | .367 | .106 | 1.274 | .114 |
| Histoloji (Nöroendokrin) | 1.147 | .231 | 5.694 | .866 |
| Diferansiasyon (İyi) |  |  |  | .316 |
| Diferansiasyon (Orta) | .585 | .259 | 1.323 | .198 |
| Diferansiasyon (Kötü) | .662 | .375 | 1.171 | .156 |
| T (T0) |  |  |  | .215 |
| T (T1) | .717 | .097 | 5.301 | .744 |
| T (T2) | 2.483 | .332 | 18.590 | .376 |
| T (T3) | .615 | .385 | .981 | .042 |
| T (T4a - T4b) | .790 | .479 | 1.302 | .356 |
| Eksize edilen LAP sayısı | 1.000 | .988 | 1.012 | .992 |
| Metastatik LAP sayısı | 1.025 | .997 | 1.054 | .082 |
| LVI (Olmayana göre olan) | .729 | .415 | 1.283 | .273 |
| PNI (Olmayana göre olan) | .648 | .358 | 1.174 | .152 |
| KRAS durumu (Wild'e göre mutant) | .851 | .603 | 1.202 | .361 |
| Metastaz zamanı (Tanı anına göre adjuvan tedavi sonrası) | .990 | .715 | 1.371 | .952 |
| Hastalıksız interval | .996 | .986 | 1.007 | .470 |
| **Metastatik bölge sayısı** | **1.375** | **1.178** | **1.604** | **.000** |
| **KC metastazı** | **.721** | **.535** | **.971** | **.031** |
| **Kemik metastazı** | **2.527** | **1.595** | **4.002** | **.000** |
| Neoadjuvantedavi (Olmayana göre olan) | .898 | .269 | 2.995 | .861 |
| Adjuvantedavi (Olmayana göre olan) | 1.062 | .757 | 1.490 | .729 |
| First-line CT (Fluoropyrimidine) |  |  |  | .007 |
| First-line CT (İrinotecan) | 2.146 | 1.296 | 3.554 | .003 |
| First-line CT (Oxaliplatin) | .931 | .678 | 1.279 | .660 |
| **First-line CT (Fluoropyrimidine'e göre kombinasyon)** | **2.196** | **1.339** | **3.600** | **.002** |
| **Tedaviye tam yanıt** | **2.648** | **1.691** | **4.147** | **.000** |

**ÇOK DEĞİŞKENLİ ANALİZ (Cox regresyon analizi) - Değişkenlerin sağkalıma etkisi**

|  | **Exp(B)** | **95.0% CI for Exp(B)** | | **Sig.** |
| --- | --- | --- | --- | --- |
|  |  | **Lower** | **Upper** |  |
| **Primer cerrahi (Uygulanmayana göre uygulanan)** | **1.406** | **1.028** | **1.924** | **.033** |
| KC metastazı | .830 | .598 | 1.152 | .265 |
| **Kemik metastazı** | **.488** | **.283** | **.840** | **.010** |
| Metastatik bölge sayısı | 1.170 | .966 | 1.416 | .108 |
| **First-line CT (Fluoropyrimidine'e göre kombinasyon)** | **1.909** | **1.140** | **3.199** | **.014** |
| **Tedaviye tam yanıt** | **2.107** | **1.324** | **3.353** | **.002** |
