# Supplementary material for: Contribution of “complete response to treatment” to survival in patients with unresectable metastatic colorectal cancer: A retrospective analysis
Source: PLoS One. 2021 Nov 8;16(11):e0259622. doi: 10.1371/journal.pone.0259622 (PMC8575296; doi:10.1371/journal.pone.0259622)
Supplement: Attachment — Submitted filename: response to reviewers.docx [file pone.0259622.s002.docx]

Response to Reviewers

İPONE-D-20-28477
Contribution of “Complete Response to Treatment” to Survival in Patients with Unresectable Metastatic Colorectal Cancer; Retrospective Analysis
PLOS ONE

Dear Dr. Editor and Reviewers;

**1. The article's titles and figure suggestions were arranged in accordance with PLOS ONE style.**

**2.The requested correction for ethical approval was rewritten in the first paragraph of the material method section.**

**3. P-values less than 0.001 was expressed as p < 0.001.**

**4. The article was edited with a professional scientific editing service.**

**5. the desired correction has been made**

**Response to the Reviewers**

1. Is the manuscript technically sound, and do the data support the conclusions?

The manuscript must describe a technically sound piece of scientific research with data that supports the conclusions. Experiments must have been conducted rigorously, with appropriate controls, replication, and sample sizes. The conclusions must be drawn appropriately based on the data presented.

Reviewer #1: Partly

**The statistical mistakes , table and explanation part of the study were corrected and correct interpretation was made.**

2. Has the statistical analysis been performed appropriately and rigorously?

Reviewer #1: No

**This article was received professional support for statistics.The statistics mistake and misinterpretations were corrected and presented.**

3. Have the authors made all data underlying the findings in their manuscript fully available?

Reviewer #1: No

**We've added the maniscpripts statistic data as part of the manuscript or its supporting information, or we've allowed to deposited to a public repository.**

4. Is the manuscript presented in an intelligible fashion and written in standard English?

Reviewer #1: No

**The article was edited with a professional scientific editing service.**

5. Review Comments to the Author

Please use the space provided to explain your answers to the questions above. You may also include additional comments for the author, including concerns about dual publication, research ethics, or publication ethics. (Please upload your review as an attachment if it exceeds 20,000 characters)

Reviewer # 1: There are multiple grammatical and typographical errors throughout the manuscript that make it difficult to easily read. These should be reviewed prior to resubmission to improve readability of manuscript. The authors performed a retrospective review of patients diagnosed with unresectable Stage IV colorectal cancer at a single institution to determine how different treatment strategies affected patient outcomes (overall survival, progression free survival), based on whether patients did or did not attain complete response (CR ) to treatment. The authors report that patients who underwent primary surgery had a higher rate of obtaining CR. Patients with liver metastasis and bone metastasis were unlikely to obtain CR. Patients with CR had longer median survival times and progression free survival. Additionally, patients who underwent first line treatment with fluoropyrimidine or irinotecan also increased overall survival, while number of metastatic regions reduced overall survival. While I believe this study and results are interesting and important, the data is presented in somewhat confusing fashion and the conclusions that are drawn are not always clearly presented. Authors should include in the abstract the type of study this was (a retrospective chart review). There are multiple places in the manuscript were it is stated p = 0.0000. While this may be correct from a rounding standpoint, from a statistical standpoint, I believe it would be more correct to state p <0.001. What exactly do the authors mean by "primary surgery"? In the Methods section, they state that any patients who underwent metastectomy during treatment were excluded. Does "primary surgery" indicate surgery as the FIRST step in their treatment? Or rather that the patient underwent surgery of their primary tumor? What surgeries did these patients undergo (resection, debulking, diversion) etc, and what were the indications for these operations? Colon and rectal cancers are grouped together in this analysis, but I do not see any mention of radiation treatment for patients with rectal cancer. Was this not included in their neoadjuvant treatment? It be interesting to see a subgroup analysis of colon vs rectal cancer to ID any difference between the two groups.

Answers;

**The article was editing by David Frances Chapman**

**The findings and discussion section presented as confusing was rewritten.**

**‘a retrospective chart review ‘ was added in abstract section**

**P-values less than 0.001 was expressed as p < 0.001.**

**Patients who received adjuvant therapy were not excluded in the study, therefore patients who underwent primary tumor resection were included in our study. This was explained in the article more clearly, necessary additions were made. Colon and rectum sub group analyzes were performed in patients and this subgroup analysis was added as a paragraph. But neoadjuvant treatment was not evaluated because only 6 of the rectal cancer patients received neoadjuvant rt. This is our limitation and was added last paragragh.**

In discussing the significant results from Table 2 - the way in which the data are presented is very confusing and the authors extrapolate new percentages within the text (albiet correct percentages based on number of patients with liver / bones mets or those who did not survive) to discuss the significant findings, but these are different than the percentages in Table 2. Would reword this to make the unders**tanding more clear**

**The percentages were rewritten correctly in the article. Mistakes ; It was presented in its corrected form.**

In discussing results from Table 6 - Since the p value is accepted as 0.05. in univariate analyzes the number of metastatic regions also both bone met and kc metastasis shortens OS time. But multivariete analises, only bone metastases were found to be statistically significant. While bone negative os contribution was significant in fig 3 according to HR, kc and numbers of metastasis region cut the line, so it was not significant.

**In univariate analysis, we realized and corrected our error presented by shortening it with the one without bone metastases and presented it correctly.**

**……….**

**How all the data in table 7 affects the OS was added to the article. the explanation was expressed more clearly.**

**………**

**Much information given with CRC with elderly patients was removed and restricted to the age paragraph only.**

In the discussion section, these results are again grouped together without delineating positive or negative association with the multiple variable discussed. The discussion session includes discussion of mCRC in elderly patients, but it is unclear to me exactly how this relates to the data presented in this study? What additional therapeutic approaches to metastatic disease are the authors referencing in the last paragraph of the Discussion section?

**The statistical mistakes , table and explanation part of the study were corrected and correct interpretation was made.Additional therapeutic approaches were actually describing sequential CT. In other words, secondary or third line treatments were not evaluated in this study. It was written into the article in a more descriptive way.**

57.2%	11.5%	12.9%	18.2%
