# Supplementary material for: Contribution of “complete response to treatment” to survival in patients with unresectable metastatic colorectal cancer: A retrospective analysis
Source: PLoS One. 2021 Nov 8;16(11):e0259622. doi: 10.1371/journal.pone.0259622 (PMC8575296; doi:10.1371/journal.pone.0259622)
Supplement: Attachment — Submitted filename: response to reviewers .docx [file pone.0259622.s003.docx]

Response to Reviewers

İPONE-D-20-28477
Contribution of “Complete Response to Treatment” to Survival in Patients with Unresectable Metastatic Colorectal Cancer; Retrospective Analysis
PLOS ONE

Dear Dr. Editor and Reviewers;

Journal Requirements:

**Response th Editor: Wanted changes in references was done.**

Reviewer's Responses to Questions

Comments to the Author

1. If the authors have adequately addressed your comments raised in a previous round of review and you feel that this manuscript is now acceptable for publication, you may indicate that here to bypass the “Comments to the Author” section, enter your conflict of interest statement in the “Confidential to Editor” section, and submit your "Accept" recommendation.

Reviewer #1: (No Response)

2. Is the manuscript technically sound, and do the data support the conclusions?

The manuscript must describe a technically sound piece of scientific research with data that supports the conclusions. Experiments must have been conducted rigorously, with appropriate controls, replication, and sample sizes. The conclusions must be drawn appropriately based on the data presented.

Reviewer #1: Yes

3. Has the statistical analysis been performed appropriately and rigorously?

Reviewer #1: Yes

4. Have the authors made all data underlying the findings in their manuscript fully available?

The PLOS Data policy requires authors to make all data underlying the findings described in their manuscript fully available without restriction, with rare exception (please refer to the Data Availability Statement in the manuscript PDF file). The data should be provided as part of the manuscript or its supporting information, or deposited to a public repository. For example, in addition to summary statistics, the data points behind means, medians and variance measures should be available. If there are restrictions on publicly sharing data—e.g. participant privacy or use of data from a third party—those must be specified.

Reviewer #1: Yes

4. Have the authors made all data underlying the findings in their manuscript fully available?

The PLOS Data policy requires authors to make all data underlying the findings described in their manuscript fully available without restriction, with rare exception (please refer to the Data Availability Statement in the manuscript PDF file). The data should be provided as part of the manuscript or its supporting information, or deposited to a public repository. For example, in addition to summary statistics, the data points behind means, medians and variance measures should be available. If there are restrictions on publicly sharing data—e.g. participant privacy or use of data from a third party—those must be specified.

Reviewer #1: Yes

5. Is the manuscript presented in an intelligible fashion and written in standard English?

**Response the review: the article was editted for standart English**

6. Review Comments to the Author

Please use the space provided to explain your answers to the questions above. You may also include additional comments for the author, including concerns about dual publication, research ethics, or publication ethics. (Please upload your review as an attachment if it exceeds 20,000 characters)

Reviewer #1: Informed consent was obtained from all 222 patients in the study? This is not typical of a retrospective chart review.

**Response the reviewer: Informed consent was obtained as it was the request of the university ethics committee. However, this information in the article has been removed to avoid confusion for the reader.**

There are still multiple grammatical and typographical errors throughout the paper that make it somewhat difficult to read, for example (this does not include all the errors in the article, just a sampling):

Abstract

Background - second sentence is an incomplete sentence

**Response the reviewer: second sentence was completed**

Results - in the sentence discussing primary surgery - what two groups are being compared?

is completion response the same as complete response? Confusing to change the verbiage

**Response the reviewer: Result section in abstract was re-written according to reviewer recommendation.**

Conclusion - mCRC is used without being previously defined in Abstract

**Response the reviewer: mCRC was defined in Abstract**

Materials and Methods

"if the primary tumor had been operated" is not correct grammar

**Response the reviewer: this sentence was written correctly**

Abbreviations are used inconsistently throughout the article. If you are using CT to represent chemotherapy, it should be that way through the entire article

**Response the reviewer: the abbreviations were written consistently by scanning the entire article.**

Overall, the introductions, methods, and results are much improved (and interesting) from the prior version.

**Response the reviewer: Thank you very much for your valuable contribution.**

The Discussion, however, remains disorganized and confusing.

The authors include information about novel anti-VEGF, anti-EGFR targeted agents, but these were not included in the study presented and it is confusing how they then tie this to their findings of longer survival times in patients who attained CR. Yes, these new agents are important to include, but I do not understand how this is related to your data?

The paragraph starting with " A total of 20,023 patients" seems completely out of order within the discussion and is not clear what first 24 clinical trials are being referenced?

Then the discussion switches back to talking about chemotherapy again.

I suggest that the authors reorganize the discussion section and ensure that they focus on their findings.

Changed paragraph places so that it can continue within a flow, and focus on our findings

**Response the reviewer: Discussion section was re-written. extra information of except findingswas removed. Targeted agents was mentioned only in the times of overall survey in discussion.**

Conclusion

What results can physicians use when counseling patients with CR?

**Response the reviewer: Wanted changes was done in conclusion.**
