# Supplementary material for: Contribution of “complete response to treatment” to survival in patients with unresectable metastatic colorectal cancer: A retrospective analysis
Source: PLoS One. 2021 Nov 8;16(11):e0259622. doi: 10.1371/journal.pone.0259622 (PMC8575296; doi:10.1371/journal.pone.0259622)
Supplement: Attachment — Submitted filename: response to reviewers 13.06.docx [file pone.0259622.s004.docx]

Response to Reviewers

İPONE-D-20-28477
Contribution of “Complete Response to Treatment” to Survival in Patients with Unresectable Metastatic Colorectal Cancer; Retrospective Analysis
PLOS ONE

Dear Dr. Editor and Reviewers;

***General comments:***

*Authors referred about metastatic colorectal cancer of two types, liver and bone metastasis. Liver metastasis is the most frequent, opposit is bone metastasis, it is one of rare metastatic site. Is some reason, why authors selected those two types?*

***1-6 metastatic sites were evaluated and included in the article. however, since these two metastatic sites are very significant, the study focused on these two sites. This comment has been added to the article.***

*The authors enrolled 222 patients and evaluated complete response  (Yes-No) in 212persons. The percentage for CR group is fitting with 212 persons (e.g. section Results first para, section Discussion, first para).*

***the number of patients is given in the article. Complete response was evaluated only in these 202 patients, as the full data of 202 patients were available.This comment has been added to the article.***

*Maybe I would recomend authors to simplify the situation and presented results only from those, who were evaluated from CR point. This is a main aim of the study. In this case Table 1 could be removed.*

***table 1 could be removed.***

*The authors omitted to mention the end of follow up period, the exact date (month/year).*

***The end of follow up date (month/year) was added the manuscript***

*Why the authors focused on two distinct group of patients? First, incidentally diagnosed metastatic CRC and second on those with developed metastasis after primary tumor resection? The second group might be associated with chemoresistance or treatment response.*

***The patient group is metastatic colorectal cancer patients. This group includes patients who have previously been operated and/or received adjuvant therapy.This comment has been added to the article.***

*128 patients had primary surgery, only 47 had adjuvant chemotherapy, please, add some comment.*

***80 patients had primary surgery but only 48 patients received adjuvant therapy. This information has also been added to the article.***

*18 patients had no LVI and 21 patients had no PNI -  does it mean that they have only liver or bone metastasis?*

***Since the initial pathology was evaluated, there was no LVI and PNI from patients who were not metastatic at the time of diagnosis. Added as a comment to the article.***

*The study belongs to rather small study, it should be mentioned in the limitation of the study.*

***This knowledge added to the limitations paragraph.***

***Particular comments***

*Abstract:*

*last sentence:  ......and the combination therapy regimens…*

***corrected in the article.***

*Material and Methods*

*to add the date of termination of follow up*

***corrected in the article.***

*Results*

*As mentioned above, Table 1 could be removed, the text and next table contain all information.*

*Table 3 is also perfectly described in the text and can be omitted. Also because the numbers for Exp(B) in case of bone metastasis are nonsense and numbers in case of fluoropyrimidine are missing.*

*In the text is abbreviation HR, in first appearance maybe should be explained (hazard ratio).*

***Table 1 and 3 was removed in manuscript***

*Table 6 - there are some empty boxes(cells) in the table, but the significance is calculated. It is confusing. Especially in the case, when is significant result (First line CT(Fluoropyrimidine)).*

*The abbreviation "LAP" is not explain*

***corrected in the article.***

*Discussion*

*First paragraph, last sentence.*

*...first line CT (combination compare with fluoropyrimidine alone)...*

*Second paragraph: The sentence "Significantly, OS and PFS outliers were lower with increasing age [13]." is not understandable. Does it mean: "OS and PFS number of outliers was significantly lower with increasing age" ?*

***corrected in the article.***

*Fourth para, fourth sentence: my suggestion how to reformulate:*

*In the study of D´Angelica et al. on 49 colorectal cancer patients with unresectable hepatic metastasis was reported that only…..*

*corrected in the article.*

*Last para*

*Interestingly, the CR was an independent predictor of survival for about 40months in OS and for about 15 months in PFS.*

***corrected in the article.***

*Fig. 1*

*Should be probably "First-line CT (combination with fluoropytimidine)" instead of "First-line CT (combination according to fluoropyrimidine)”*

***corrected in the article.***

Journal Requirements:

Please review your reference list to ensure that it is complete and correct. If you have cited papers that have been retracted, please include the rationale for doing so in the manuscript text, or remove these references and replace them with relevant current references. Any changes to the reference list should be mentioned in the rebuttal letter that accompanies your revised manuscript. If you need to cite a retracted article, indicate the article’s retracted status in the References list and also include a citation and full reference for the retraction notice.

**We review our reference list**

[Note: HTML markup is below. Please do not edit.]

Reviewers' comments:

Reviewer's Responses to Questions

**Comments to the Author**

Reviewer #2: I received this manuscript after the first revision. Even though the article has been revised, my feelings are confused. The article focused on determining the complete response in the metastatic CRC. However, everything that the authors studied in their study has long been known, and the conclusions of the study do not contribute much to the already acquired knowledge about the survival of metastatic CRC patients.

**Sorry for your comment. There are very few studies on complete response.**

**I have been waiting for a year for the article to be published in your journal. I'm starting to think I've been lingering for a long time**

The authors stated that CR was less common in the presence of liver metastasis and bone metastasis. My question is: If authors focused on mCRC and no lung or other distant metastasis were observed, what is it outcome of this observation?

**In this study, all metastatic areas were examined. It was evaluated from 1 to 6 metastatic sites. but there was significant results in both bone and liver. At the same time, the importance of the number of metastatic sites was mentioned in the study.**

It is not clear whether authors focused on sporadic CRC?

**Patients who developed both denovo metastatic and follow-up metastases were included in the study**.

What was the end of the study, I mean when the follow up terminated?
 **At the end of the study, patients with complete responses were found to have a long survey.**

Why the authors focused on two distinct group of patients?

**These are not two different groups, they are all metastatic patients; whether it is metastasis at the time of diagnosis or metastasis develops during follow-up.**

First, incidentally diagnosed metastatic CRC and second on those with developed metastasis after primary tumor resection?

The second group might be associated with chemoresistance or treatment response.

**Added as a comment to the article.**

Is it correct the metastasis interval? 5.93±12.81 (Mean ±SD)

**This is correct.**

The authors stated that there were 20 survivors and median patients follow up was in range 0.3 to almost 132 months. With no defined end of the study, this has no reason to show up.
If I understand correctly, 9% of those patients with no primary surgery reached CR while 42% with no primary surgery did not reach CR.

**The importance of primary surgery was mentioned in many places in the article.**

This outcome is clear from Table 2 and further again elaborated in Table 3, 6 and 7. Why it can not be only supplemented by additional text only?

**Table 1 and 3 was removed.**

Besides, 128 patients had primary surgery however only 47 had adjuvant therapy. Why? T his should be mentioned.

**Added as a comment to the article.**

Further, 18 patients had no LVI and 21 no PNI, are there really all patients in the study in stage IV?

**Added as a comment to the article.’ Because only devo metastatic patients were not evaluated’**
The main limitation is also relatively small population size that might results many of outcomes as false positives. This should be commented.

**Added as a comment to the article.**

Discussion is wordy and not focused on obtained results.
In addition, the article is written in very poor English and the several sentences do not make sense.
The article contains unnecessarily many tables and the following text, which comment on everything that can be found in the table and usually comments on insignificant results. Tables 3, 4 and 5 can be omitted and only stated by additional text as it is already.

**The article’s english was edited**

Sayın editör ;

1 yıldır belli toplam 3 defa revizyonda tüm hakemlerin isteğini yerine getirdik. Son 2 revizyon minor revizyon olup giderek daha olumsuz yorum almaya başladım bu değerlendirmenin son olmasını talep ediyorum.
