# Supplementary material for: Contribution of “complete response to treatment” to survival in patients with unresectable metastatic colorectal cancer: A retrospective analysis
Source: PLoS One. 2021 Nov 8;16(11):e0259622. doi: 10.1371/journal.pone.0259622 (PMC8575296; doi:10.1371/journal.pone.0259622)
Supplement: Attachment — Submitted filename: response to reviewers 22.08.docx [file pone.0259622.s005.docx]

Response to Reviewers

Dear Editor,

ABSTRACT:
  In addition, to is evaluate progression-free survival…– should be: In addition, to evaluate progression-free survival…
… In subgroup patients with who underwent primary surgery; the number….should be: In subgroup patients who underwent primary surgery, the number…

***the necessary changes was made.**

RESULTS: 
The sentence: Because only devo metastatic patients were not evaluated; should be deleted

Twenty (9.0%) patients were still alive, 222 (91.0%) patients had died .- it is some mistake or typing error, this does not correspond with total number of enrolled patients (222=100%).

**Twenty (9.0%) patients were still alive, 202 (91.0%) patients had died *the necessary changes was made.**

PFS analysis, based on whether a CR was attained is shown in Table 5 
should be . PFS analysis, based on whether a CR was attained is shown in Table 3 ***the necessary changes was made.**

Table 4: 
there are missing values for Exp(B) and 95%CI in the case of: Primary region (Rectum). Histology (Adeno), Differentiation (Good) – all of them are not significant values, it is not so critical, but there are missing also in the case of First-line CT (Fluoropyrimidine), which are significant P=0.007.
Row with T0 has no sense.

**Since the number of cases in the sub group is insufficient, the values mentioned in the table cannot be given. The number of patients with a complete response in the first-line CT (Fluoropyrimidine) group is only “1”.**

**This information has been added as a table description.**

**The missing values in the table was filled and Row with T0 was deleted**

First-line CT (Combination according to Fluoropyrimidine) _ I guess should be: (Combination with Fluoropyrimidine) ***the necessary changes was made.**

Table 5
First-line CT (Combination according to Fluoropyrimidine) _ I think it should be: (Combination with Fluoropyrimidine). ***the necessary changes was made.**

DISCUSSION:
The sentence: However, the vast majority (80-90%) present with unresectable diseases should be:
However, the vast majority (80-90%) presents unresectable diseases ***the necessary changes was made.**

The sentence:
…..whereas it is approximately 2 years in those treated with a combination of irinotecan or oxaliplatin to fluoropyrimidine-based CT.  
Should be:… whereas it is approximately 2 years in those treated with a combination of irinotecan or oxaliplatin with fluoropyrimidine-based CT. ***the necessary changes was made.**

FIGURE 3:
First-line CT (Combination according to Fluoropyrimidine) _ should be: Combination with Fluoropyrimidine
Decreases in Number of metastatik lesion 
should be: Decreases in Number of metastatic lesions

***the necessary changes was made.**
